# Clock Gene Variants Are Associated with Energy and Macronutrient Intake in Early Childhood and Adulthood

**DOI:** 10.3390/nu18121906

**Published:** 2026-06-12

**Authors:** Zachary J. Ribau, Sanjeena Subedi, Lori Ann Vallis, Hannah J. Coyle-Asbil, Angela Annis, Madeline Nixon, Lyn Hillyer, Alison M. Duncan, Jess Haines, David W. L. Ma

**Affiliations:** 1 Department of Human Health Sciences, College of Biological Science, University of Guelph, Guelph, ON N1G 2W1, Canada; zribau@uoguelph.ca (Z.J.R.);; 2 School of Mathematics and Statistics, Faculty of Science, Carleton University, Ottawa, ON K1S 5B6, Canada; 3 School of Occupational Therapy, Faculty of Health Sciences, University of Western Ontario, London, ON N6A 3K7, Canada; 4 Department of Family Relations and Applied Nutrition, College of Social & Applied Human Sciences, University of Guelph, Guelph, ON N1G 2W1, Canada

**Keywords:** circadian genetics, clock gene polymorphisms, dietary behaviour, childhood obesity, nutrigenetics, circadian rhythm, pediatric nutrition

## Abstract

**Background/Objectives:** Obesity remains a global health concern, and personalized prevention strategies that consider genetic predispositions can enhance existing strategies. Research suggests that variation in circadian rhythm-related genes, or clock genes, may influence obesity risk, in part through effects on dietary behaviour. However, associations between single-nucleotide polymorphisms (SNPs) in clock genes and dietary outcomes remain understudied, particularly in children. Therefore, we investigated cross-sectional associations between clock gene SNPs and dietary outcomes using baseline data from 226 adults (138 females, 88 males) aged 26–50 y and 168 children (90 females, 78 males) aged 2–6 y from the Guelph Family Health Study. **Methods:** DNA was extracted from saliva and genotyped using the Illumina Global Diversity Array, and dietary intake was assessed using the Automated Self-Administered 24 h Dietary Assessment Tool. Nine SNPs representing 8 clock genes were selected based on prior associations with dietary and obesity-related outcomes. Generalized Estimating Equations were used to test associations, adjusted for multiple comparisons with the Benjamini–Hochberg false discovery rate (FDR) procedure. **Results:** Ten nominal associations were identified (*p* < 0.05), and 2 remained significant after FDR correction (P_adj_ < 0.05); among children, rs2314339-T (*NR1D1*) was associated with a lower percentage of energy from protein (β = −2.4%, P_adj_ = 0.003) and rs11605924-A (*CRY2*) with higher energy intake (β = 118.0 kcal, P_adj_ = 0.044). **Conclusions:** Findings suggest that clock gene SNPs may influence dietary habits from early childhood. Future longitudinal and functional studies are needed to clarify whether these variants can inform precision nutrition strategies for obesity prevention.

## 1. Introduction

Obesity continues to pose significant public health challenges [1,2,3], with multifaceted environmental and genetic factors contributing to its rise. Dietary intake represents a core, modifiable component of obesity risk [4,5]; consequently, substantial research has been dedicated to understanding the regulation of dietary behaviours. Emerging evidence has pointed to circadian rhythm-related factors, including sleep [6,7] and meal timing [8], sleep regularity [9], and variation in related genes [10,11,12,13], commonly referred to as clock genes, as potential regulators of dietary behaviours. Clock genes are of particular interest due to a multitude of experimental studies supporting their influence on feeding behaviour [14,15,16], energy homeostasis and metabolism [17,18,19,20,21], as well as obesity and related phenotypes [19,22,23]. Further, single-nucleotide polymorphisms (SNPs) in several clock genes have been observationally linked to obesity [24,25,26,27,28,29,30,31,32,33,34,35] and parameters related to obesity and Type 2 Diabetes (T2D) [36,37,38,39,40] in diverse human populations, including individuals of European, East Asian, African, and South Asian ancestry, and in both adults and children. Together, research suggests that clock genes may contribute to obesity risk, at least in part, by influencing dietary behaviours. Clock gene SNPs may therefore represent candidate markers for the early identification of obesity risk, and help to inform personalized, diet-based interventions. Despite this, few studies have examined clock gene SNPs in relation to dietary patterns in pediatric populations, and, to the best of our knowledge, no studies have been conducted in children younger than 6 years old.

### 1.1. The Clock Genes

The circadian system synchronizes internal processes with external cues, primarily the light-dark cycle. A central pacemaker in the suprachiasmatic nucleus (SCN) receives light input through the retina and aligns the phase of peripheral clock components throughout the body, thereby coordinating rhythmic behaviours such as sleep and feeding [41]. Molecularly, these rhythms are driven by the clock genes, which operate in an autoregulatory transcription-translation loop in which one of clock circadian regulator (CLOCK) or neuronal PAS domain protein 2 (NPAS2) heterodimerizes with basic helix-loop-helix ARNT like 1 (BMAL1) to activate transcription of the period circadian regulators 1-3 (*PER1*, *PER2*, *PER3*) and cryptochrome circadian regulators 1 and 2 (*CRY1*, *CRY2*). The resulting PER and CRY proteins dimerize to form PER:CRY complexes, which accumulate and progressively inhibit CLOCK:BMAL1 activity, generating self-sustaining molecular oscillations that approximate 24 h periods in humans. Given their tight coupling and widespread physiological influence throughout the body, clock genes have garnered substantial interest as modulators of disease risk.

### 1.2. Clock Genes, Dietary Outcomes and Obesity

Extensive observational and some experimental evidence associate clock gene variation with an array of obesity- and diet-related outcomes. In adults, cross-sectional and case–control studies have associated *CLOCK* variants with overweight and obesity risk [24,25,26,33], differences in total energy or macronutrient intake [10,13,42], and with both dietary and obesity-related outcomes [27,43]. Among the most widely studied is the rs1801260 polymorphism, an A>G substitution with a global minor allele frequency of approximately 26% [44], with the minor variant associated with obesity and BMI [26,27], waist-to-hip ratio [33], and circadian rhythm and sleep phenotypes associated with a higher risk of obesity [33,45,46]. However, research on specific dietary phenotypes in relation to obesity risk remains limited and inconsistent [47]. Variants in other clock genes have also been studied in adults, including rs228729 and a Variable Number Tandem Repeat (VNTR) polymorphism of *PER3*, linked to an extreme obesity phenotype [30] and energy derived from carbohydrate and fat [12], respectively. SNPs in *PER2* were associated with abdominal obesity, obesogenic eating behaviours, and a greater likelihood of attrition in a diet-based weight reduction intervention study [31], while SNPs in *CRY1* and *CRY2* were associated with insulin resistance [36], fasting glucose concentrations [37,40,48,49,50], and increased risk of T2D [40,49]. Additionally, rs2314339-T of nuclear receptor subfamily 1 group D member 1 (*NR1D1*) has been inversely associated with adiposity measures [32,33], highlighting the need to characterize the contributions of individual SNPs to complex, polygenic phenotypes.

Although limited, some studies have evaluated *CLOCK* gene variation in children and adolescents in relation to adiposity and, to a lesser extent, dietary behaviours. As in adults, the most studied SNP is rs1801260-G; however, SNPs in *BMAL1* and *NR1D1* have also been associated with obesity and related anthropometric measures in children and adolescents [29,34]. Across five studies that examined rs1801260-G in children of ages ranging from 6 to 14 years, three found no significant association with BMI or BMI z-score [51,52,53], one reported a positive association in females, but not in males [28], and one found a positive association between meal frequency and BMI z-score, without a direct genetic effect [11]. Notably, one of these studies reported a female-specific association for a different *CLOCK* variant, rs4864548-A, with BMI [53]. Sex-dependent clock gene-adiposity associations may reflect broader sex differences in circadian biology [54,55,56], as well as parental feeding practices and attitudes that may affect the way children eat prior to puberty [57]. Interestingly, another study demonstrated differences in *PER3* CpG site methylation between children with obesity and healthy controls [58], pointing to epigenetic regulation as an additional vector through which clock genes may contribute to obesity phenotypes. Collectively, evidence linking clock genes to obesity and related outcomes in children remains heterogeneous, and no studies have examined these associations in children under 6 years of age. Since the development of obesity is complex and occurs over time, it may be difficult to detect subtle genetic influences in early childhood. By contrast, genetically influenced appetite traits, which may manifest through dietary measures, may be more readily detectable and serve as potential intermediate phenotypes linking clock genes to obesity risk in early life.

Existing evidence and strong biological plausibility suggest that clock genes may influence appetite and dietary behaviour, energy homeostasis, and the development of obesity and related complications, including T2D. However, the relationships between circadian biology, dietary behaviour, and obesity are likely bidirectional [41,59], which makes causal interpretation difficult to establish. To identify variants relevant for clinical application, an important first step is to characterize associations between specific polymorphisms and dietary outcomes, particularly in pediatric populations where these associations remain understudied. Therefore, the aim of this study was to investigate associations between clock gene SNPs and dietary outcomes in children aged 2 to 6 years old and their parents from the Guelph Family Health Study (GFHS) cohort. We hypothesized that clock gene SNPs previously linked with obesity, T2D, and dietary outcomes would be associated with differences in total energy and macronutrient-derived energy intake in both adults and children. This research extends prior studies of clock gene SNPs by examining these associations in a novel pediatric cohort, contributing to a growing understanding of how clock genes influence dietary behaviour and relate to obesity risk from early life.

## 2. Methods

### 2.1. Participants and Data Collection

Baseline data were obtained from 243 families, including 385 adults and 283 children, enrolled in the full study cohort of the GFHS, which is an ongoing family-focused obesity prevention programme initiated in 2017. Families from the Guelph-Wellington area with at least one child aged 2 to 6 years were recruited. The GFHS aims to better understand early life risk factors for obesity, with a focus on translating research findings into actionable prevention strategies. The University of Guelph Research Ethics Board approved the study (REB#17-07-003). Additional details on the GFHS are described elsewhere [60].

Dietary, anthropometric, demographic, and health-related data were collected from adults and children of the GFHS from March 2016 to July 2024. Dietary data were obtained through completion of 24 h dietary intake recalls using the Canadian version of the Automated Self-Administered 24 h Dietary Assessment Tool (ASA24) [61]. Adults completed the dietary recall online for themselves and for their children participating in the study. Participant height and body mass were measured by trained staff during baseline health assessment visits, and used to calculate BMI (adults) and BMI z-score (children). Household income, parental education, ethnicity, chronic health conditions, and self-reported (adults) and parent-reported (children) weekly hours of moderate and vigorous physical activity were acquired from baseline surveys.

Genetic data were obtained from saliva samples collected from May 2017 to September 2019. DNA was extracted from 2 mL of saliva using the DNA Genotek saliva collection kit (DNA Genotek Inc., Ottawa, ON, Canada; Cat# OGD-610) according to the manufacturer’s instructions. Briefly, PrepIT-L2P (DNA Genotek, Cat# PT-L2P-5) was used for purification, and DNA concentration and purity were determined using Agilent TapeStation 4150 (Agilent Technologies Canada Inc., Mississauga, ON, Canada). All samples had a DNA integrity number (DIN) over 7.0. 500 ng of DNA was sent to The Centre for Applied Genomics Microarray Facility (SickKids Hospital, Toronto, ON, Canada) for SNP analysis using the Illumina Human Global Diversity Array (GDA) 8 (v1.0) platform capable of detecting 1.9 million SNPs. Additional details are available in Supplemental Text S1.

### 2.2. Selection of Target SNPs

Relevant SNPs were identified from prior studies reporting associations between clock gene SNPs and dietary, metabolic, or anthropometric outcomes related to obesity. Priority was given to SNPs significantly associated with dietary measures or obesity, and to associations reported in human clinical trials. Where an identified SNP was not available in the GFHS genotype array, proxy variants found to be in linkage disequilibrium (LD) were identified using a threshold R^2^ statistic ≥ 0.8, using the *LDlinkR* package (version 1.4.0) [62] for the R programming language (version 4.5.3) [63]. After a set of target SNPs was identified and their presence confirmed in the genotype array, pairwise LD was assessed for all SNPs located on the same chromosome. Pairs found to be in LD (R^2^ ≥ 0.8) were ranked, and only a single representative SNP was retained. All LD calculations were based on representative population data from the 1000 Genomes Project, independently confirmed within each of the European (EUR), East Asian (EAS), and South Asian (SAS) superpopulations [64].

### 2.3. Data Processing

Participants’ data were retained for the analysis if their records were complete across the required demographic, anthropometric, dietary, and genetic variables. These included age, sex, BMI or BMI z-score, educational attainment, ethnicity, a complete baseline ASA24 food recall, and valid genotypes for all target SNPs. Adults who were pregnant or breastfeeding were not included in the analysis. Survey responses for household income and educational attainment were standardized to a single reporting parent (the first parent to enrol in each family), for use as covariates in the children’s cohort. Where household income was not reported, values were imputed with the sample mean, calculated separately within adults and children, and before any filtering measures were taken. Energy contributions from carbohydrate, protein, and fat were calculated by multiplying their respective gram amounts by standard Atwater conversion factors [65]: 4 kcal per g for carbohydrate and protein, and 9 kcal per g for fat.

Participant genotypes for all target clock gene SNPs were obtained by matching standard SNP identification numbers (rsIDs) to loci included in the genotyping array. SNP associations are typically reported relative to an effect allele, but determining true allelic orientation requires knowledge of both the design probe and reference genome strand orientations, which vary by SNP in modern genotyping arrays. This is particularly important for strand-ambiguous SNPs (A/T or C/G), especially when the study population allele frequencies are near 50% and cannot be used for strand inference. For consistency, we standardized all SNP alleles to the plus (+) strand using annotations from the Illumina manifest (v1.0 D1, GRCh37), and flipped alleles according to Illumina’s TOP/BOT rules. Mappings were then converted to GRCh38.p14 to align with current genomic standards. Population frequency statistics were then computed independently in adults and in children, including allelic and genotypic frequencies. At this stage, SNPs were removed from the analysis if the minor allele comprised < 5% of the total allele pool. To ensure directional comparability of effect estimates, reference and effect allele assignments were aligned to select comparative studies for each SNP. Genotypes were numerically encoded by mapping 0, 1 or 2 to the genotype combinations derived from the reference and effect allele assignments. Either additive or dominant genetic models were used, depending on genotype group frequencies. An additive model was used when the lowest-frequency genotype group accounted for ≥5% of the population. In additive models, 0 was assigned for the homozygous reference, 1 for the heterozygous, and 2 for the homozygous effect-allele genotypes. For SNPs where the lowest-frequency genotype group represented <5% of the population, a dominant model was used to preserve statistical power; accordingly, 0 indicated the homozygous reference genotype, and 1 indicated both other genotypes.

### 2.4. Statistical Analysis

All statistical analyses and visualizations were performed in R. SNP-outcome association analyses were conducted separately for adults and children. Associations were tested for each SNP with energy intake and with the percentage of energy from carbohydrate, protein, and fat individually as the response. For each dietary outcome, separate models were fitted for each SNP, adjusting for covariates specific to the outcome and cohort. Age, sex (female, male), household income (midpoint of reported range), educational attainment (1 = Some high school; 2 = High school graduate; 3 = Some college or technical school; 4 = College graduate; 5 = Some university; 6 = University graduate; 7 = Postgraduate training or degree), ethnicity (White, Non-white), and either BMI (adults) or BMI z-score (children) were included as covariates in the energy intake and each macronutrient energy model for both adults and children. Note, educational attainment was modelled linearly as a numeric variable; alternative coding schemes were tested in sensitivity analyses. In adults, additional covariates in energy intake models included total mg of caffeine, which has been shown to reduce appetite [66], as well as the presence of one or more common health conditions that may influence daily energy intake, encoded as a binary categorical variable (Y/N), including Ulcerative Colitis, Bulimia, Crohn’s disease, Irritable Bowel Syndrome, and Depression.

Generalized Estimating Equations (GEE) were used in both adults and children to account for non-independence of observations arising from familial relatedness and shared household environments, implemented in R with the *geepack* package (version 1.3.12) [67]. Family identification codes were used as the clustering parameter, and an exchangeable correlation structure was specified under an assumption of consistent correlation among individuals within the same family. All outcome variables were visually evaluated for approximate normality to support the assumption of a Gaussian distribution, which was specified for all models. *p*-values were derived from Wald statistics and adjusted for multiple comparisons using the Benjamini–Hochberg false discovery rate (FDR) procedure, applied to each set of tests conducted for a given outcome within each cohort. Significance was determined at an adjusted *p*-value (P_adj_) of < 0.05. Beta (β) coefficients and the corresponding 95% confidence intervals (CI) are reported for association tests reaching nominal significance (*p* < 0.05). Boxplots were used to visualize the distributions of outcomes for associations that remained significant after FDR correction, grouped by genotype of the associated variant.

Secondary and sensitivity analyses were also conducted to examine potential sex differences and sample bias due to the inclusion criteria, and to evaluate whether results were sensitive to the inclusion of BMI and BMI z-score, weekly hours of physical activity, or to the coding scheme used for educational attainment. To evaluate potential sex differences, all models were refitted with a SNP × sex interaction term. Sex-specific and interaction β coefficients, 95% CIs, and *p*-values were derived from the resulting models using estimated marginal trends; FDR correction was applied to all *p*-values generated for a given outcome. To evaluate bias due to inclusion criteria, differences in baseline demographic characteristics were compared between participants in the final analytical sample and those excluded using t-tests for age, BMI and child BMI z-score; Mann–Whitney U tests for household income and educational attainment; and chi-square tests for sex, ethnicity, and in adults, the presence of one or more common health conditions that may influence energy intake. To evaluate the influence of missing household income handling on the study results, all SNP association analyses were repeated in a sample where participants with missing household income values were excluded, rather than using mean-imputed values. Moreover, models were refitted with educational attainment collapsed to 4 levels (1 = No post-secondary; 2 = College graduate; 3 = University graduate; 4 = Postgraduate training or degree) and treated as either categorical or ordinal variables to compare against the initial numeric encoding with 7 levels. Note: Educational attainment is collapsed to 4 levels for reporting in descriptive characteristics tables. Finally, all models were refitted after excluding BMI and BMI z-score and, separately, after including weekly hours of physical activity, to examine the sensitivity of results to these covariates.

## 3. Results

### 3.1. Participant Characteristics

A total of 394 participants from 164 families were included in the final analytical sample, comprising 168 children and 226 adults, after exclusion due to a lack of genetic data (*n* = 240) and missingness in required demographic or anthropometric variables (*n* = 34). The children’s dataset contained 28 pairs of siblings and one trio of siblings. In the adults’ dataset, there were 72 participant pairs (2 parents) with all remaining individuals from independent families. Participant characteristics are shown for adults and children in Table 1 and Table 2. Those included in the final analytical sample did not significantly differ in demographic variables from those excluded, except for age, which was lower among excluded adults (*p* = 0.032) and children (*p* = 0.008).

### 3.2. Clock Gene SNPs Included

The final set of SNPs analyzed, and their genotype frequencies in the adults’ and children’s cohorts, are shown in Table 3. None of the selected SNPs located on the same chromosome were found to be in LD, and all SNPs had minor allele frequencies exceeding 5%. Additionally, all selected SNPs are non-coding and therefore do not result in amino acid sequence changes. In both cohorts, rs2304672, rs3816358, and rs2314339 were analyzed with dominant genetic models.

### 3.3. SNP-Diet Associations

**Figure 1:**
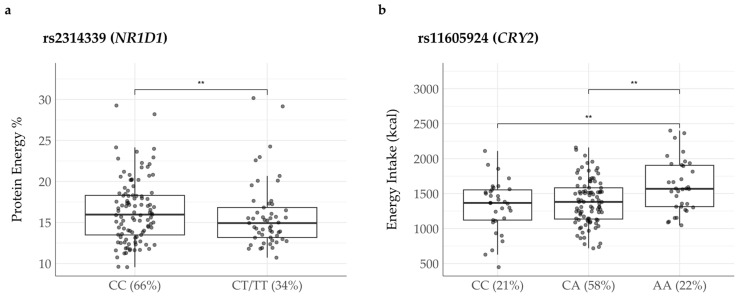
Distribution of dietary outcomes of the two lead SNP associations stratified by genotype: (**a**) percentage of energy from protein by genotype for rs2314339 (*NR1D1*; homozygous reference allele CC, heterozygous or homozygous effect allele CT/TT) and (**b**) energy intake by genotype for rs11605924 (*CRY2*; homozygous reference allele CC, heterozygous CA, homozygous effect-allele AA) in children; frequencies are shown in parentheses. For rs2314339 (panel (**a**)), the CT and TT genotypes are collapsed under a dominant model. Asterisks indicate significant pairwise comparisons based on estimated marginal means from the respective Generalized Estimating Equation models, Tukey-adjusted. Significance: ** *p* < 0.01.

Ten SNP-diet associations reached nominal significance (*p* < 0.05), involving 6 different SNPs; moreover, 2 remained significant after applying FDR correction (Table 4). These included rs2314339-T (*NR1D1*) with lower percentage of energy from protein (β = −2.4%, P_adj_ = 0.003) and rs11605924-A (*CRY2*) with higher energy intake (β = 118.0 kcal, P_adj_ = 0.044) in children. The distributions of dietary outcomes for the two lead SNP associations stratified by genotype are shown in Figure 1. Notably, a nominal association was also observed for rs2314339-T with a higher percentage of energy from carbohydrate (β = 3.1%, *p* = 3.1 × 10^−2^) in children.

Additional associations reaching nominal significance in children included rs228729-T (*PER3*) with a lower percentage of energy from carbohydrate (β = −2.7%, *p* = 0.007) and a higher percentage of energy from fat (β = 1.9%, *p* = 0.033), in addition to rs3816358-A (*BMAL1*) with a lower percentage of energy from carbohydrate (β = −3.3%, *p* = 0.029). In adults, nominal associations included rs1801260-G (*CLOCK*) with lower energy intake (β = −205.3 kcal, *p* = 0.022), rs2304672-C (*PER2*) with higher energy intake (β = 410.8 kcal, *p* = 0.034) and percentage of energy from fat (β = 3.5%, *p* = 0.035), and rs228729-T with a lower percentage of energy from protein (β = −1.0%, *p* = 0.049).

In a secondary analysis, we tested the statistical homogeneity of all effects by sex in the regression models with an interaction term. All interaction effects are reported relative to the reference group (sex = female). No sex differences were observed for the two lead associations identified in the main analysis. In children, nominally significant SNP × sex interactions were observed for the associations of rs2314339-T (*NR1D1*) (β_interaction_ = 4.9%, P_interaction_ = 0.016), rs1801260-G (β_interaction_ = −3.6%, P_interaction_ = 0.031), and rs4864548-A (*CLOCK*) (β_interaction_ = 3.2%, P_interaction_ = 0.035) with percentage of energy from fat (Supplemental Table S1). Within this outcome, nominally significant associations were observed in females, but not in males, for rs2314339-T (β = −3.2%, *p* = 0.048) and rs1801260-G (β = 2.6%, *p* = 0.011). In adults, one significant SNP × sex interaction was observed for the association of rs2314339-T with energy intake (β_interaction_ = −565.3 kcal, P_interaction_ = 0.001), with a nominally significant within-sex association observed in males only (β = −352.8 kcal, *p* = 0.009). Distributions of dietary outcomes split by genotype and sex for all associations involving a SNP × sex interaction term reaching nominal significance are shown in Supplemental Figure S1.

Sensitivity analyses showed that the main analysis results were not meaningfully changed when excluding observations with missing household income values (*n* = 17) instead of using mean imputation. When educational attainment was re-coded to 4 levels and treated as categorical (instead of numeric), the statistical significance of some associations changed. Two associations in children, previously identified as nominal in the main analysis, were significant after adjusting for multiple testing: rs228729-T and rs2314339-T with lower and higher percentages of energy from carbohydrate, respectively. When models were refitted with (i) exclusion of BMI and BMI z-score and (ii) inclusion of weekly hours of physical activity as covariates, the statistical significance, direction, and magnitude of the FDR-corrected significant findings were not substantively changed. Several associations no longer reached nominal significance: among children, rs2314339-T with a higher percentage of energy from carbohydrate under (i), and rs228729-T with a higher percentage of energy from fat under (ii); among adults, rs2304672-C with higher energy intake under (i), and rs228729-T with a lower percentage of energy from protein under (i) and (ii). Two additional nominal associations emerged under (ii): rs2304672-C and rs3816358-A, with a higher percentage of energy from protein in children.

## 4. Discussion

In this study, we examined cross-sectional associations of 9 genetic variants of clock genes with total and macronutrient-derived energy intake in both adults and, for the first time, children under 6 years of age. Of 10 SNP-diet associations reaching nominal significance in adults and children, 2 remained significant after FDR correction: rs2314339-T (*NR1D1*) with a lower percentage of energy from protein and rs11605924-A (*CRY2*) with higher energy intake, both observed in children. Overall, these findings complement prior observational [31,32,33] and clinical studies [38] on the SNPs examined, and provide novel evidence of clock gene-diet effects manifesting in young children.

The most robust association identified in this study was between rs2314339-T of *NR1D1* and a lower percentage of energy from protein in children. While protein intake alone is unlikely to contribute substantially to obesity risk, a recent systematic review found that higher total protein intake during early childhood was associated with higher BMI in later childhood and adolescence, with stronger evidence for this association with animal-based compared to plant-based protein [68]. Notably, Garaulet et al. [32] found significantly lower rates of abdominal obesity in adult carriers of rs2314339-T in two independent Mediterranean (*n* = 1465; mean age 39.4 years) and North American (*n* = 820; mean age 48.6 years) cohorts. Interestingly, the authors also reported higher levels of physical activity among those with rs2314339-T, and no differences in energy intake. Similarly, Molina-Montes et al. [33] reported a lower rate of long-term weight gain in males with rs2314339-T in the European Prospective Investigation into Cancer and Nutrition (EPIC)-Spain chronodiet study (*n* = 2505; mean age 42.6 at recruitment to 65.3 years at follow-up), though only at the nominal level. At the gene level, several other *NR1D1* variants have been associated with measures of adiposity in adolescents [34,35], and experimental work has highlighted potential appetite-related pathways linking *NR1D1* to adiposity. Mice deficient in REV-ERBα, the protein encoded by *NR1D1*, have increased hypothalamic orexin signalling and heightened motivation for palatable food [69], as well as impaired leptin sensitivity, contributing to increased feeding and weight gain [70]. Thus, evidence suggests that rs2314339-T may influence obesity risk, likely involving a combination of activity and appetite-related mechanisms that may evolve with age. The association of rs2314339-T with lower protein in young children that we observed may reflect an early manifestation of an appetite-modifying effect. Future research should validate this finding and investigate whether rs2314339-T is associated with lower rates of obesity in children, with consideration of physical activity and additional confounding dietary factors.

A significant association was also observed between rs11605924-A of *CRY2* and higher energy intake in children. Previous observational studies of rs11605924-A have focused on T2D and robustly link the polymorphism to higher fasting glucose [37,40,48,49,50], consistent with experimental evidence in mice demonstrating that depletion of the CRY proteins increases hepatic mRNA levels for gluconeogenic genes and raises circulating glucose compared with controls [71]. Higher energy intake has been associated with insulin resistance and fasting glucose in children aged 9–10 years [72], effects only partly attenuated by adjustment for fat mass index. This suggests that higher total energy intake from fats and carbohydrates may contribute to T2D risk through mechanisms partially independent of adiposity, such as pancreatic β-cell glucolipotoxicity [73]. In this context, our finding may indicate an early, contributory dietary signal for rs11605924-A, detectable upstream of established risk factors for T2D and obesity; however, fasting glucose, T2D, and obesity were not evaluated in this analysis. Alternatively, dietary factors may act as modifiers of rs11605924-associated T2D risk, which may help explain why, despite well-established associations with fasting glucose and strong mechanistic plausibility, rs11605924-A has not been consistently associated with T2D risk across all populations studied [74]. Evidence from a 2-year diet-based clinical trial by Mirzaei et al. [38] suggests that dietary fat intake may modify rs11605924-associated differences in energy expenditure, albeit in the context of an intervention targeting overweight and obesity (*n* = 721; mean age 51 years). These researchers found that rs11605924-A predicted greater increases in resting energy expenditure at 2 years than at baseline, with significantly larger effects in adults randomized to a high-fat compared with a low-fat diet [38]. Mechanistic inference from a large study by Machicao et al. [48] suggests a potential explanation for how dietary moderation of rs11605924-associated metabolism may influence T2D. In their study (*n* = 1715; mean age 39 years), rs11605924-A was associated with higher fasting glucose and, counterintuitively, with lower hepatic fat content in a sub-sample of participants who underwent hepatic magnetic resonance spectroscopy. They further reported that *CRY2* mRNA expression was positively associated with hepatic triglyceride synthesis, estimated in a smaller sub-sample of tissue donors. This suggests that impairment of *CRY2* function reduces triglyceride synthesis while increasing gluconeogenesis, providing a plausible physiological explanation for the metabolic phenotypes associated with rs11605924-A. Viewed in this context, higher energy intake in children with rs11605924-A may reflect secondary metabolic demands associated with altered energy metabolism; however, energy expenditure was not measured in this analysis and should be included in future studies. Additionally, further investigation is required to determine if dietary macronutrient distribution moderates rs11605924-associated risk of T2D, with potential implications for personalized nutrition strategies targeting T2D prevention.

Additional nominal associations warrant discussion. In adults, the G allele of rs1801260 was nominally associated with lower energy intake. Among six prior studies examining rs1801260 in relation to energy intake, only one reported a significant association, whereby the G allele was associated with higher energy intake [46,47]. Moreover, a recent exploratory analysis in young adult males (*n* = 30, mean age 27.7 years) found a nominal association between the G allele and reduced postprandial cravings suppression after a standardized meal [75]. Our nominal finding, therefore, adds to a heterogeneous literature for rs1801260 and dietary phenotypes, highlighting the need for standardized methodological approaches and adjustment for cohort characteristics and environmental factors in future studies [47]. Additionally, we identified nominal associations between the C allele of rs2304672 and higher fat-derived and overall energy intake among adults, consistent with a prior study from Garaulet et al. [31] reporting that carriers of rs2304672-C (G in their study, see Appendix A) displayed more frequent snacking, stress-related eating, and higher attrition rates in weight-loss programmes among adults with overweight or obesity (*n* = 239; mean age 39.2 years). Interestingly, a neuroimaging study by Forbes et al. [76] reported that carriers of rs2304672-C (G in their study, see Appendix A) had reduced medial prefrontal cortex activity during reward processing, a neural process involved in response to highly palatable foods [77]. Taken together, prior research suggests that rs2304672-C may promote behavioural patterns associated with higher obesity risk, and our data extend prior associations to higher total and fat-derived energy intake. However, these associations did not remain significant after FDR correction, and should therefore be considered exploratory.

In children, rs228729-T was nominally associated with a lower percentage of energy from carbohydrate and a higher percentage of energy from fat. In adults, the T allele was associated with a lower percentage of energy from protein. These findings, particularly concerning the dietary pattern observed in children for rs228729-T, are consistent with a prior study linking the long (5-repeat) allele of the *PER3* VNTR with higher fat and lower carbohydrate intake in a sample of European adults who completed seven-day food records (*n* = 329, mean age 46.9 years) [12]. A dietary pattern consisting of lower carbohydrate and higher fat content is not inherently obesogenic, as the origin and type of these nutrients are also relevant; however, these findings suggest that dietary macronutrient preference may underlie prior *PER3*-obesity associations in adults and children. For instance, Azevedo et al. [30] found that rs228729-T was significantly associated with a higher probability of obesity and extreme obesity in adults, with the latter classified as having a BMI ≥ 40. In children, a genome-wide methylation analysis (*n* = 24; mean age = 10.6) from Samblas et al. [58] found an inverse correlation between *PER3* CpG site methylation and BMI z-score, suggesting that epigenetic reductions in *PER3* expression may contribute to adiposity in early life. Future research should investigate the functional consequences of specific *PER3* polymorphisms in relation to long-term weight trajectories, with a particular emphasis on clarifying the role of dietary patterns.

While secondary analyses did not indicate sex differences for the two lead associations identified in the main analysis, nominally significant SNP × sex interactions were observed in the associations of rs1801260, rs4864548, and rs2314339 with percentage of energy from fat in children. Furthermore, a significant interaction was observed for rs2314339-T with energy intake in adults, with a nominal inverse association observed in males and no effect in females. Our findings in children are consistent with prior reports of sex differences in the *CLOCK* gene variants in relation to BMI z-score [28,53], supporting the notion that early sex differences in *CLOCK*-associated dietary patterns may be related to sex differences in adiposity risk in later childhood. While these findings may reflect sex differences in circadian biology, it is possible that unmeasured psychosocial factors, particularly sex bias in food-parenting [57], may modify the associations reported herein. Our adult findings may reflect more pronounced differences in sex hormones in adulthood relative to early childhood, which may contribute to sex-dimorphic circadian gene expression [55] and SCN organization and signalling [54]. However, our finding contrasts with the previously discussed study by Garaulet et al. [32], which examined energy intake directly and found no differences overall or in either sex between rs2314339 genotypes. This discrepancy may reflect differences in data collection methodology, cohort characteristics, and study power. Notably, Garaulet et al. reported a lower rate of abdominal obesity and higher physical activity among carriers of rs2314339-T, proposing physical activity rather than dietary intake as a potential mechanism. Overall, our secondary analysis indicates potential sex differences in the associations of SNPs in *CLOCK* and *NR1D1* with dietary outcomes in children and adults; however, these findings are exploratory. Further work is required to confirm these associations and explore their relevance for obesity risk.

### 4.1. Sleep and Circadian Influence

Our findings advance the notion that clock genes are related to dietary intake. While not specifically assessed in our work, clock gene-related variability in sleep and circadian rhythm patterns is likely to influence dietary outcomes and obesity risk alongside the mechanisms discussed to this point. For instance, circadian preference, widely referred to as chronotype, describes an individual’s tendency toward earlier or later sleep timing, and is determined in part by clock gene variation [45,78]. Individuals who tend toward later sleep and wake times often have higher rates of obesity [79], a relationship that may be explained by dietary behaviours such as later meal timing and increased evening energy intake [80,81], and more frequent snacking on energy-dense foods [82]. Similarly, habitual short sleep duration, which has been epidemiologically linked to obesity [83], has been associated with several clock genes [45,84]. Studies show that sleep deprivation can increase leptin and reduce ghrelin the following day [85,86], promote hedonic eating [87], and increase overall energy intake [88,89]. Reduced sleep duration may also promote increased energy consumption, as a longer waking period provides an extended window for eating. While energy expenditure may also increase with longer waking periods, it has been suggested that energy consumption increases to a greater extent, which results in a positive energy balance [90]. Considering these relationships, future research is needed to clarify if clock gene-diet associations are better understood through sleep and circadian behaviours, or through appetite and metabolism-related mechanisms.

### 4.2. Strengths and Limitations

This study has several strengths. Most notably, we examined clock gene SNPs and dietary outcomes in children aged 2–6 years, advancing our understanding of circadian gene-diet interactions in early stages of development, when early dietary habits may contribute to later disease risk. Other strengths of this study include robust data collection methodologies, namely the validated ASA24 dietary assessment tool, and objectively measured height and body mass used to derive BMI and BMI z-scores. Finally, this study employed a rigorous methodological approach to the analysis of a targeted set of clock gene SNPs, including the use of additive or dominant genetic modelling on a per-SNP basis, handling within-family clustering with GEE, and controlling for false discovery rate.

This analysis also has several limitations to consider. Firstly, this was a cross-sectional analysis that evaluated dietary parameters from a single 24 h dietary recall, which is prone to recall and reporting bias, and limits the robustness of habitual intake estimation. Additionally, the dietary outcomes examined may have been influenced by unmeasured, circadian-related factors, including chronotype and objective measures of physical activity and sleep. Moreover, meal timing and diet quality represent important dietary outcomes that were not examined in this study and warrant investigation in future research. Another limitation is that most participants were of white European ancestry, which limits generalizability to other populations where genotype frequencies for the studied SNPs often differ substantially. Additionally, statistical power to detect SNP × sex interactions was limited by small genotype subgroups and an imbalanced sex ratio, particularly among adult participants (male-to-female ratio = 0.64). Further, because children between 2 and 6 years old are not entirely in control of the type and quantity of food they consume, parental influence is likely to affect the dietary outcomes examined. While this was partially addressed by including family identification code as the clustering parameter in the children’s GEE models, this does not comprehensively account for parental influences on children’s dietary behaviours. Finally, only two associations identified from the main analysis remained significant after FDR correction, reflecting modest effect sizes and a need for validation in larger, independent cohorts. As dietary behaviour is a multifactorial and likely polygenic phenotype, the contribution of any single variant is unlikely to inform personalized nutrition recommendations, limiting any immediate clinical applicability of these findings.

### 4.3. Future Perspectives

Replication of these findings in independent cohorts is needed, particularly in larger, ethnically diverse cohorts. Additionally, future longitudinal studies should aim for comprehensive circadian phenotyping, including chronotype, sleep duration and quality, meal timing, and physical activity. Gene-environment interactions should also be examined, particularly those involving early childhood food environment and sleep routines. Such research will help clarify the contribution of circadian gene variants to obesity risk across stages of childhood and beyond.

Functional studies are also warranted to characterize the influence of the examined SNPs on circadian gene expression, and to elucidate the neuroendocrine mechanisms linking clock gene variants to appetite regulation and energy balance. Additionally, the identification of key biological pathways can be advanced through multi-omics approaches, including metabolomics, transcriptomics, epigenetics, and microbiome analyses. As SNPs typically exert only modest effects in isolation, especially with respect to complex behavioural phenotypes such as eating behaviour, future efforts should evaluate their combined effects to support the potential development of polygenic risk scores. Collectively, such research will help clarify the potential contribution of circadian genetic profiling to precision nutrition strategies.

## 5. Conclusions

Overall, we present novel associations between clock gene SNPs and dietary outcomes in adults and, for the first time, in children as young as 2 to 6 years of age. SNPs in *CLOCK*, *PER2*, and *CRY2* were associated with energy intake, whereas *NR1D1*, *BMAL1*, *PER2*, and *PER3* were associated with macronutrient-derived energy contributions. Of the ten associations reaching nominal significance, 2 remained significant after FDR correction: rs2314339-T (*NR1D1*) with a lower percentage of energy from protein, and rs11605924-A (*CRY2*) with higher energy intake, both observed among children. Our findings build on prior studies of the examined SNPs, contributing to a growing body of evidence linking circadian genetics to dietary behaviour, an important modifier of obesity risk. Furthermore, our data suggest that clock gene variants may influence dietary behaviour from early childhood. Future longitudinal and functional studies are needed to validate these associations and ultimately clarify the potential for clock gene variants to inform precision nutrition strategies for early obesity prevention.

## Figures and Tables

**Table 1:** Adults’ cohort characteristics.

Characteristic	Overall	Female	Male
*n*	226	138	88
Age in years, mean (SD)	36.0 (4.3)	35.4 (4.2)	36.8 (4.4)
BMI, mean (SD)	26.8 (6.1)	26.3 (6.6)	27.5 (5.1)
Ethnicity, *n* (%)			
Non-White	42 (18.6)	23 (16.7)	19 (21.6)
White	184 (81.4)	115 (83.3)	69 (78.4)
Education, *n* (%)			
Some university, some college or technical school, or less	33 (14.6)	13 (9.4)	20 (22.7)
College graduate	34 (15.0)	22 (15.9)	12 (13.6)
University graduate	72 (31.9)	43 (31.2)	29 (33.0)
Postgraduate training or degree	87 (38.5)	60 (43.5)	27 (30.7)
Household income ($CA) ^1^, *n* (%)			
<$60,000	33 (14.6)	21 (15.2)	12 (13.6)
$60,000–$99,999	64 (28.3)	42 (30.4)	22 (25.0)
$100,000–$149,999	80 (35.4)	46 (33.3)	34 (38.6)
>$150,000	49 (21.7)	29 (21.0)	20 (22.7)
Dietary Intake, mean (SD)			
Energy (kcal)	2254.9 (851.6)	2075.5 (744.7)	2536.2 (933.5)
% Energy from Fat	36.8 (9.0)	37.1 (8.6)	36.3 (9.8)
% Energy from Protein	17.0 (5.1)	17.1 (5.3)	16.8 (4.9)
% Energy from Carbohydrate	45.9 (10.3)	46.6 (10.3)	44.9 (10.2)

^1^ Household income was imputed with the adult sample mean for 17 observations. Abbreviations: SD, standard deviation; BMI, body mass index.

**Table 2:** Children’s cohort characteristics.

Characteristic	Overall	Female	Male
*n*	168	90	78
Age in years, mean (SD)	3.6 (1.2)	3.6 (1.2)	3.7 (1.2)
BMI z-score, mean (SD)	0.5 (0.8)	0.5 (0.8)	0.6 (0.8)
Ethnicity, *n* (%)			
Non-White	34 (20.2)	16 (17.8)	18 (23.1)
White	134 (79.8)	74 (82.2)	60 (76.9)
Parent Education ^1^, *n* (%)			
Some university, some college or technical school, or less	14 (8.3)	9 (10.0)	5 (6.4)
College graduate	23 (13.7)	10 (11.1)	13 (16.7)
University graduate	54 (32.1)	31 (34.4)	23 (29.5)
Postgraduate training or degree	77 (45.8)	40 (44.4)	37 (47.4)
Household income ($CA) ^1^, *n* (%)			
<$60,000	27 (16.1)	15 (16.7)	12 (15.4)
$60,000–$99,999	47 (28.0)	27 (30.0)	20 (25.6)
$100,000–$149,999	51 (30.4)	25 (27.8)	26 (33.3)
>$150,000	43 (25.6)	23 (25.6)	20 (25.6)
Dietary Intake, mean (SD)			
Energy (kcal)	1414.7 (358.5)	1396.1 (354.5)	1436.2 (364.2)
% Energy from Fat	33.8 (7.1)	34.4 (6.6)	33.1 (7.7)
% Energy from Protein	16.4 (4.2)	16.8 (4.2)	15.9 (4.1)
% Energy from Carbohydrate	51.8 (8.7)	50.8 (8.1)	52.9 (9.3)

^1^ Parental education and household income reported by Parent 1. Abbreviations: SD, standard deviation; BMI, body mass index.

**Table 3:** Target SNP characteristics and genotype frequencies, *n* (%).

			Adults			Children		
Gene	rsID	Alleles (Aa) ^1^	AA	Aa	aa	AA	Aa	aa
*CLOCK*	rs1801260	AG	121 (54%)	89 (39%)	16 (7%)	93 (55%)	62 (37%)	13 (8%)
	rs4864548	GA	94 (42%)	96 (42%)	36 (16%)	70 (42%)	69 (41%)	29 (17%)
*CRY1*	rs2287161	GC	65 (29%)	107 (47%)	54 (24%)	54 (32%)	76 (45%)	38 (23%)
*CRY2*	rs11605924	CA	59 (26%)	100 (44%)	67 (30%)	34 (20%)	94 (56%)	40 (24%)
*NPAS2*	rs4850954	TC	41 (18%)	122 (54%)	63 (28%)	46 (27%)	72 (43%)	50 (30%)
*NR1D1*	rs2314339	CT	166 (73%)	50 (22%)	10 (4%)	112 (67%)	49 (29%)	7 (4%)
*PER2*	rs2304672	GC	196 (87%)	30 (13%)	0 (0%)	147 (88%)	21 (12%)	0 (0%)
*BMAL1*	rs3816358	CA	164 (73%)	59 (26%)	3 (1%)	128 (76%)	36 (21%)	4 (2%)
*PER3*	rs228729	CT	96 (42%)	103 (46%)	27 (12%)	77 (46%)	77 (46%)	14 (8%)

^1^ Reference (A) and effect (a) alleles at each locus, assigned based on prior literature to ensure directional comparability of associations. AA, Aa and aa represent the homozygous reference, heterozygous, and homozygous effect-allele genotypes, respectively.

**Table 4:** Summary of SNP-diet associations reaching nominal significance (*p* < 0.05) in the adults’ and children’s cohort analyses.

Cohort	Effect Variant ^1^	Gene	Outcome	β (95% CI) ^2^	*p*	*P*_adj_ ^3^
Adults	rs1801260-G	*CLOCK*	Energy Intake	−205.3 (−380.8, −29.8)	2.2 × 10^−2^	1.5 × 10^−1^
	rs2304672-C	*PER2*	Energy Intake	410.8 (30.2, 791.4)	3.4 × 10^−2^	1.5 × 10^−1^
	rs2304672-C		Fat Energy %	3.5 (0.2, 6.7)	3.5 × 10^−2^	2.7 × 10^−1^
	rs228729-T	*PER3*	Protein Energy %	−1.005 (−2.007, −0.003)	4.9 × 10^−2^	4.4 × 10^−1^
Children	rs2314339-T *	*NR1D1*	Protein Energy %	−2.4 (−3.7, −1.1)	3.4 × 10^−4^	3.3 × 10^−3^
	rs11605924-A *	*CRY2*	Energy Intake	118.0 (35.9, 200.1)	4.9 × 10^−3^	4.4 × 10^−2^
	rs228729-T	*PER3*	CHO Energy %	−2.7 (−4.6, −0.7)	6.6 × 10^−3^	6.0 × 10^−2^
	rs3816358-A	*BMAL1*	CHO Energy %	−3.3 (−6.2, −0.3)	2.9 × 10^−2^	9.4 × 10^−2^
	rs2314339-T	*NR1D1*	CHO Energy %	3.1 (0.3, 5.9)	3.1 × 10^−2^	9.4 × 10^−2^
	rs228729-T	*PER3*	Fat Energy %	1.9 (0.1, 3.6)	3.3 × 10^−2^	3.0 × 10^−1^

^1^ SNP identification number (rsID) and effect allele. ^2^ Estimated regression coefficient (β) and 95% CI per effect allele. ^3^ Benjamini–Hochberg FDR-adjusted *p* values. * Denotes significance (adjusted *p* < 0.05). Abbreviations: SNP, single nucleotide polymorphism; CI, confidence interval; FDR, false discovery rate; CHO, carbohydrate.

## Data Availability

Interested researchers can contact GFHS investigators to explore data availability in alignment with the University of Guelph Research Ethics Board.

## Supplementary Materials

The following supporting information can be downloaded at: https://www.mdpi.com/article/10.3390/nu18121906/s1, Text S1: DNA extraction; Figure S1: Distributions of dietary outcomes of SNP associations with nominally significant SNP × sex interaction terms stratified by sex and genotype; Table S1: Summary of SNP × sex interaction tests reaching nominal significance in the adults’ and children’s secondary analyses.

## Appendix A

**Table A1:** Allele-reporting comparisons for strand-ambiguous SNPs in prior studies.

		Cited Study		This Study	
rsID	Study	Alleles (Aa) ^1^	% Aa/aa ^2^	Alleles (Aa) ^1^	% Aa/aa ^2^
rs2304672	Garaulet et al. [31]	CG	12.6	GC	12.5
	Forbes et al. [76]	CG	13.3		

^1^ Reference (A) and effect (a) nucleotides as reported by cited studies (left) and this study (right). ^2^ Frequency of the combined heterozygous and homozygous effect-allele genotypes in cited studies (left) compared to this study (right).

In the absence of confirmed strand orientation, an inference can be made for the ambiguous rs2304672 variant based on the reported genotype frequencies; since the variant allele is relatively uncommon, it is reasonably distinguishable by frequency.
