# Supplementary material for: Clock Gene Variants Are Associated with Energy and Macronutrient Intake in Early Childhood and Adulthood
Source: Nutrients. 2026 Jun 12;18(12):1906. doi: 10.3390/nu18121906 (PMC13305929; doi:10.3390/nu18121906)
Supplement: Supplementary file 1 [file nutrients-18-01906-s001.zip › nutrients-4331835-supplementary.pdf]

Supplementary Materials

Text S1. *DNA extraction*

DNA was extracted from 2 mL of saliva using the DNA Genotek saliva collection kit (DNA Genotek, Cat# OGD-610) according to the manufacturer's instructions. Samples were mixed by inversion and incubated overnight in a 50°C water bath. Next day, the samples were transferred to a 15 mL conical centrifuge, volume was recorded and PrepIT-L2P (DNA Genotek, Cat# PT-L2P-5) was added at 1/25 the original measured volume. Samples were vortexed and incubated on ice for 10 min. and then centrifuged at 3500 x g for 20 min. The supernatant was transferred to a fresh 15 mL conical centrifuge tube, the volume was measured, and 100% ethanol was added at 1.2 times the measured volume. Samples were gently mixed by inversion and incubated at room temperature for 15 min. and then centrifuged for 20 min. at 3500 x g. The supernatant was carefully removed to avoid disturbing the DNA pellet. 1 mL of 70% ethanol was carefully added to the pellet and incubated at room temperature for 1 min. The ethanol was removed and the DNA was rehydrated by the addition of 500 uL Tris-EDTA, vortexed and incubated overnight at room temperature. Next day, samples were transferred to a 1.5 mL microcentrifuge tube and 5 uL of 10ug/mL ribonuclease A (Sigma, Cat # R4875) and 2uL of 25 U/mL ribonuclease T1 (Sigma, Cat# R1003) were added. Samples were incubated at 37°C for 30 min. after which, 10uL of 0.1M sodium chloride was added followed by 1 mL of 95% ethanol. Samples were incubated at room temperature for 10 min. then centrifuged at a speed of 13000 x g for 2 min. The supernatant was discarded and the DNA pellet was redissolved in 50uL Tris-EDTA buffer. DNA concentration and purity were determined by Agilent Tapestation 4150.

Table S1. Summary of SNP × sex interaction tests reaching nominal significance in the adults' and children's secondary analyses.

		Female		Male		Interaction	
**Effect Variant[1]**	**Outcome**	**β[2]**	***p***	**β[2]**	***p***	**β[2]**	***p***
**Adults**							
rs2314339-T	Energy Intake	212.6 (-3.6, 428.7)	5.4×10^{-2}	-352.8 (-615.5, -90.0)	8.7×10^{-3}	-565.3 (-905.3, -225.3)	1.1×10^{-3}
**Children**							
rs1801260-G		2.6 (0.6, 4.6)	1.1×10^{-2}	-1.0 (-3.5, 1.5)	4.3×10^{-1}	-3.6 (-6.9, -0.3)	3.1×10^{-2}
rs2314339-T	Fat Energy %	-3.2 (-6.3, -0.0)	4.8×10^{-2}	1.8 (-1.6, 5.1)	3.0×10^{-1}	4.9 (0.9, 9.0)	1.6×10^{-2}
rs4864548-A		-1.5 (-3.5, 0.6)	1.7×10^{-1}	1.8 (-0.5, 4.0)	1.2×10^{-1}	3.2 (0.2, 6.2)	3.5×10^{-2}

[1] SNP identification number (rsID) and effect allele. [2] Estimated sex-specific and interaction regression coefficients (β) and 95% CI derived from the GEE models using estimated marginal trends. Abbreviations: SNP, single nucleotide polymorphism; CI, confidence interval; GEE, Generalized Estimated Equations.

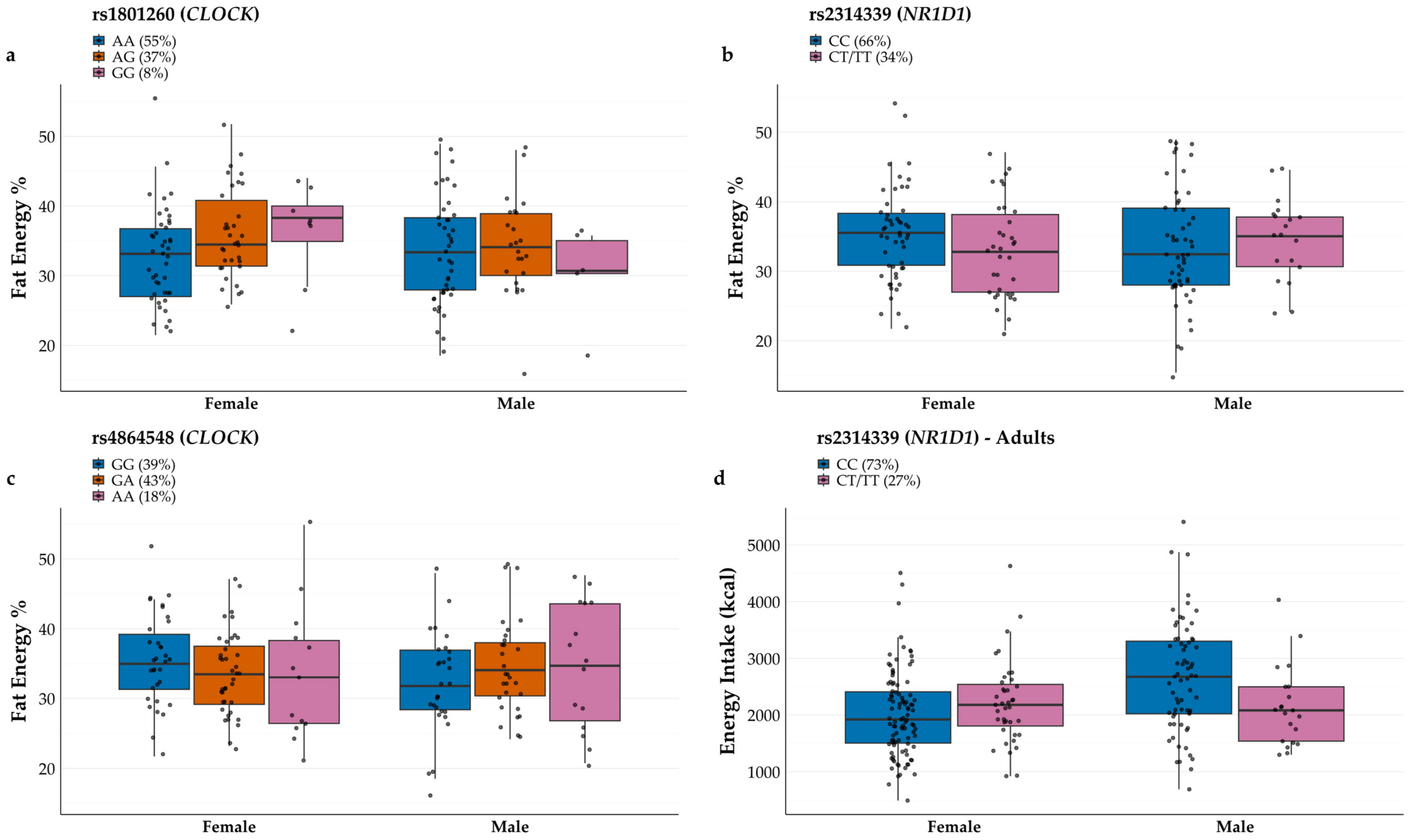

Figure S1. Distributions of dietary outcomes of SNP associations with nominally significant SNP × sex interaction terms stratified by sex and genotype: **(a)** percentage of energy from fat by sex and genotype for rs1801260 (*CLOCK*; homozygous reference allele AA, heterozygous AG, homozygous effect allele GG), **(b)** rs2314339 (*NR1D1*; homozygous reference allele CC, heterozygous or homozygous effect allele CT/TT) and **(c)** rs4864548 (*CLOCK*; homozygous reference allele GG, heterozygous GA, homozygous effect allele AA) in children, and **(d)** energy intake by sex and genotype for rs2314339 in adults. Genotype groups are indicated by colour and ordered from left to right by effect allele dosage; frequencies are shown in parentheses relative to the overall cohort (both sexes combined). For rs2314339 (panels b and d), the CT and TT genotypes are collapsed under a dominant model.
